# Coenzyme Q10 Levels Are Decreased in the Cerebellum of Multiple-System Atrophy Patients

**DOI:** 10.1371/journal.pone.0149557

**Published:** 2016-02-19

**Authors:** Lucia V. Schottlaender, Conceição Bettencourt, Aoife P. Kiely, Annapurna Chalasani, Viruna Neergheen, Janice L. Holton, Iain Hargreaves, Henry Houlden

**Affiliations:** 1 Department of Molecular Neuroscience, UCL Institute of Neurology, Queen Square, London, United Kingdom; 2 Department of Clinical and Experimental Epilepsy, UCL Institute of Neurology, Queen Square, London, United Kingdom; 3 Reta Lila Weston Institute for Neurological Studies and The Queen Square Brain Bank, London, United Kingdom; 4 Neurometabolic Unit, National Hospital, London, United Kingdom; 5 Neurogenetics Laboratory, The National Hospital for Neurology and Neurosurgery, Queen Square, London, United Kingdom; 6 The MRC Centre for Neuromuscular Diseases, UCL Institute of Neurology, London, United Kingdom; Hertie Institute for Clinical Brain Research and German Center for Neurodegenerative Diseases, GERMANY

## Abstract

**Background:**

The objective of this study was to evaluate whether the levels of coenzyme Q10 (CoQ10) in brain tissue of multiple system atrophy (MSA) patients differ from those in elderly controls and in patients with other neurodegenerative diseases.

**Methods:**

Flash frozen brain tissue of a series of 20 pathologically confirmed MSA patients [9 olivopontocerebellar atrophy (OPCA) type, 6 striatonigral degeneration (SND) type, and 5 mixed type] was used for this study. Elderly controls (n = 37) as well as idiopathic Parkinson's disease (n = 7), dementia with Lewy bodies (n = 20), corticobasal degeneration (n = 15) and cerebellar ataxia (n = 18) patients were used as comparison groups. CoQ10 was measured in cerebellar and frontal cortex tissue by high performance liquid chromatography.

**Results:**

We detected a statistically significant decrease (by 3–5%) in the level of CoQ10 in the cerebellum of MSA cases (P = 0.001), specifically in OPCA (P = 0.001) and mixed cases (P = 0.005), when compared to controls as well as to other neurodegenerative diseases [dementia with Lewy bodies (P<0.001), idiopathic Parkinson's disease (P<0.001), corticobasal degeneration (P<0.001), and cerebellar ataxia (P = 0.001)].

**Conclusion:**

Our results suggest that a perturbation in the CoQ10 biosynthetic pathway is associated with the pathogenesis of MSA but the mechanism behind this finding remains to be elucidated.

## Introduction

Multiple system atrophy (MSA) is a fatal late onset neurodegenerative condition. Clinically, MSA is characterized by parkinsonism, cerebellar ataxia and autonomic dysfunction. Pathologically, it presents with widespread alpha-synuclein immunoreactive glial cytoplasmic inclusions and neurodegeneration [1], and can be subdivided into 3 neuropathological subtypes according to the affected brain regions. These subtypes are: 1) olivopontocerebellar atrophy (OPCA), 2) striatonigral degeneration (SND), and 3) a combination of the two, referred to as mixed [2]. These subtypes correspond to the clinical subdivision of MSA into MSA-C (OPCA), MSA-P (SND) and mixed. MSA is often classified as an alpha-synucleinopathy together with idiopathic Parkinson’s disease (IPD) and Dementia with Lewy bodies (DLB) in view of the intracellular accumulation of alpha-synuclein. The etiology of MSA is still unknown although a genetic component has been proposed [3–6].

Coenzyme Q10 (CoQ10) is a lipophilic molecule located in the inner mitochondrial membrane and functions as an electron transporter from complexes I and II to complex III in the respiratory chain [7]. CoQ10 is the predominant coenzyme Q in humans, has an antioxidant role, transports electrons in extramitochondrial systems and participates in the regulation of the mitochondrial transition permeability pore [7].

Complete CoQ10 knockout in mammalian models has been shown to be embryonic lethal [8, 9]. In humans, disorders of CoQ10 deficiency are subdivided into primary CoQ10 deficiencies in which pathogenic mutations occur in the genes required for CoQ10 biosynthesis or secondary caused by mutations in genes not involved in biosynthesis (reviewed in [10]). The phenotype of these patients is usually different from the clinical presentation of MSA patients [11]. CoQ10 deficiencies present as mitochondrial diseases, symptoms including encephalomyopathy, ataxia with cerebellar atrophy and can also involve renal failure. The onset of CoQ10 deficiency generally occurs in childhood, although there are a few cases of onset in early adulthood [12]. CoQ10 deficiency constitutes a very interesting and potentially treatable condition with CoQ10 supplements, and is thought to be inherited in an autosomal recessive fashion.

*COQ2* is a gene that encodes for parahydroxybenzoate-polyprenyl transferase, an enzyme that participates in the synthetic pathway of CoQ10. Through linkage analysis and whole-genome sequencing, variants in *COQ2* have been linked to clinically diagnosed Japanese MSA patients in a multicenter study. This study also described decreased growth rates in *COQ2* mutant yeasts and proposed that variants in the *COQ2* gene are a cause of familial MSA and a risk factor for sporadic MSA [6]. We and other groups have been unable to replicate this genetic finding so far [13–15]. The authors of that study, by analyzing a small sample group, suggested decreased levels of CoQ10 in brain tissue of MSA cases (n = 3) in comparison with controls (n = 3)[6]. In view of this result, we decided to determine the level of CoQ10 in brain tissue from a large cohort of pathologically confirmed MSA cases and compare these levels to those of normal controls as well as to patients with other neurodegenerative movement disorders.

## Materials and Methods

### Standard protocol approvals and informed consent

University College London Hospitals (UCLH) ethics committee approved the study (UCLH ethics approval UG2UPD|04/Q0505/2). Brain tissue was obtained from the Queen Square Brain Bank (QSBB), UCL and the London Neurodegenerative Diseases Brain Bank, Institute of Psychiatry, King’s College London, in London (UK), the MRC Sudden Death Brain and Tissue Bank, in Edinburgh (UK), the IDIBAPS Brain Bank, in Barcelona (Spain), the Human Brain and Spinal Fluid Resource Center, in Los Angeles (USA), and the Harvard Brain Tissue Resource Center, in Belmont MA (USA). Written informed consent was obtained from all participants. Tissue stored in the QSBB is under a license from the Human Tissue Authority and has been donated for research according to protocols approved by the NRES Committee London-Central.

### Subjects

We analyzed the available pathologically confirmed MSA cases (n = 20) and pathologically normal elderly controls (n = 37). With the purpose of comparing to other alpha-synucleinopathies we also analyzed DLB cases (n = 20) and IPD cases (n = 7) and to compare to a tauopathy we used corticobasal degeneration (CBD) cases (n = 15). Finally, to compare to other degenerative diseases that affect the cerebellum we decided to include cerebellar ataxia (CRB_ATX) cases (n = 18). A description of the cases used in this study is given in Table 1 [16]. We used flash frozen brain tissue from these cases and we measured CoQ10 in the cerebellum (cerebellar cortex) and frontal cortex (Brodmann areas 8 or 9). Cerebellum was selected as a severely affected region in MSA, particularly in OPCA and mixed pathological subtypes, and frontal cortex as an overall less affected brain region in this disease. Basal ganglia regions, which would be more severely affected in MSA-SND pathological subtype, were not available in most cases and were therefore excluded. All MSA cases included in this study have been sequenced for the entire coding region of the *COQ2* gene [14], with no *COQ2* variants associated with increased risk of MSA being found in the entire cohort.

**Table 1:** Characterization of the samples included in this study.

Diagnosis groups	MSA all	MSA-OPCA	MSA-MIX	MSA-SND	CBD	DLB	IPD	CRB_ATX^a^	Controls
**Demographic features**									
Number of cases	20	9	5	6	15	20	7	18	37
Cases with CRBL available	20	9	5	6	15	20	7	18	37
Cases with FCTX available	20	9	5	6	0	20	7	18	28
Gender (% of female)	70%	56%	80%	83%	40%	25%	14%	50%	46%
Mean age at death, years (range)	64.55 (51; 74)	64.1 (57; 72)	65.4 (57; 73)	64.5 (51; 74)	69.60 (48; 90)	77.72 (66; 92)	77.86 (65; 84)	59.89 (36; 88)	81.32 (63; 102)
Mean post-mortem delay, hours	53.44	44.96	67.86	54.14	53.43	21.23	54.89	33.25	15.66
**Unadjusted levels of CoQ10 (pmol/mg)**									
Mean CRBL (±SD)	169.30* (±49.71)	150.52** (±29.12)	163.44** (±73.40)	202.33 (±41.79)	271.18 (±76.21)	288.37 (±133.72)	262.47 (±28.84)	233.08 (±46.97)	241.87 (±57.70)
Mean FCTX (±SD)	260.44 (±70.22)	264.87 (±75.19)	283.98 (±88.91)	234.17 (±44.12)	-	256.94 (±75.20)	276.02 (±71.37)	330.12 (±96.14)	259.39 (±107.09)

*MSA presents significantly lower cerebellar CoQ10 levels when compared to all other diagnosis groups (Tukey test, p ≤ 0.002)

**when dividing by disease subgroups, only MSA-OPCA and MSA-Mix show significantly lower levels than controls, CBD, DLB, FRDA^a^, and IPD (Tukey test, p ≤ 0.02); no other significant differences were observed between groups for CoQ10 levels.

Glossary: MSA = multiple system atrophy; MSA-OPCA = MSA olivopontocerebellar atrophy; MSA-MIX = MSA mixed type; MSA-SND = MSA striatonigral degeneration; CBD = corticobasal degeneration; DLB = dementia with Lewy bodies; IPD = idiopathic Parkinson’s disease; CRB_ATX = cerebellar ataxia [^a^includes: SCA = spinocerebellar ataxia (n = 9); FRDA = Friedreich's ataxia (n = 5); other ataxias (miscelaneous) (n = 4)]; CRBL = cerebellar cortex; FCTX = frontal cortex; SD = standard deviation.

### Brain tissue homogenization and CoQ10 measurement

Flash-frozen brain tissue (including similar amounts of grey and white matter) was homogenized with a buffer that contained sucrose, EDTA and Tris (tris[hydroxymethyl]aminomethane). The PH was 7.4 and the buffer was isotonic with the brain cells. Forty μL of the homogenates were mixed with an internal standard (IS). The IS used was di-propoxy-CoQ10 which is a synthetic non-physiological ubiquinone. The IS was added to the brain homogenates which were then vortex mixed, frozen in liquid nitrogen, and thawed twice to ensure maximal release of cellular CoQ10. A solvent of hexane:ethanol (70:30% (v/v)) was added to the sample, vortex mixed for 1 minute, and then centrifuged (5 min x 14,000 g, 25°C). Following centrifugation, the upper organic layer containing lipophilic compounds including CoQ10 was removed and evaporated to dryness. The sample was reconstituted in ethanol and loaded onto a HPLC which was linked to a UV detector set at 275 nm. Ubiquinones, including CoQ10 have a characteristic absorbance at 275 nm and the HPLC is calibrated with a quantified CoQ10 and IS prior to analyzing the biological samples. This method is described in detail elsewhere [17].

### Statistical analyses

Data were analyzed with SPSS (v.22). For both cerebellar and frontal cortex tissue, one-way analysis of variance was performed to check for omnibus significant differences in the CoQ10 levels between the main diagnosis groups (MSA, CRB_ATX, CBD, DLB, IPD, and controls). A post hoc Tukey HSD test was then used for pairwise comparisons. To reach a normal distribution of the CoQ10 levels, this variable was log-transformed prior to the abovementioned analyses. These analyses were also performed considering subgroups for MSA (MSA-OPCA, MSA-Mix and MSA-SND) and CRB_ATX (SCA, FRDA and other ataxias). Additionally, multinomial logistic regression was used to infer the magnitude of the association between the outcome (diagnosis) and levels of CoQ10, corrected for potential confounding factors. Controls were used as the reference group, except for comparisons between disease groups only; in this case MSA was taken as the reference (Table 2). All models were adjusted for age (continuous), gender (binomial), and post-mortem delay (continuous). For all the analyses performed, a p-value <0.05 was considered significant.

**Table 2:** Multinomial logistic regression estimates for the association between different diagnosis groups and CoQ10 levels in human brain tissue.

**A) All disease groups versus controls in the Cerebellum tissue**		
**Diagnosis**	**OR (95% CI)**	**p value**
**MSA**	**0.97 (0.95–0.99)**	0.001*
**CBD**	1.01 (1.00–1.02)	0.223
**DLB**	1.01 (1.00–1.02)	0.058
**IPD**	1.01 (1.00–1.02)	0.405
**CRB_ATX**	1.00 (0.99–1.01)	0.935
**B) MSA and CRB_ATX subdivided into their respective subtypes versus controls in the Cerebellum tissue**		
**Diagnosis**	**OR (95% CI)**	**p value**
**MSA_SND**	0.98 (0.96–1.01)	0.128
**MSA_Mixed**	**0.96 (0.93–0.99)**	0.005*
**MSA_OPCA**	**0.95 (0.92–0.98)**	0.001*
**SCA**	0.99 (0.97–1.01)	0.147
**FRDA**	1.01 (1.00–1.03)	0.062
**other_ataxias**	1.00 (0.97–1.02)	0.885
**C) All other degenerative diseases versus MSA in the Cerebellum tissue**		
**Diagnosis**	**OR (95% CI)**	**p value**
**CBD**	**1.04 (1.02–1.06)**	<0.001*
**DLB**	**1.04 (1.02–1.06)**	<0.001*
**IPD**	**1.04 (1.02–1.06)**	<0.001*
**CRB_ATX**	**1.03 (1.01–1.05)**	0.001*
**D) All disease groups versus controls in the Frontal cortex tissue**		
**Diagnosis**	**OR(95% CI)**	**p value**
**MSA**	1.00 (1.00–1.01)	0.846
**DLB**	1.00 (1.00–1.01)	0.792
**IPD**	1.00 (1.00–1.01)	0.543
**CRB_ATX**	1.01 (1.00–1.01)	0.123

* and **bold** highlight significant values (p<0.05). All models were adjusted for age, gender, and post-mortem delay.

Glossary: MSA = multiple system atrophy; MSA_SND = MSA striatonigral degeneration; MSA_mixed = MSA mixed; MSA_OPCA = MSA olivopontocerebellar atrophy; CBD = corticobasal degeneration; DLB = dementia with Lewy bodies; IPD = idiopathic Parkinson’s disease; CRB_ATX = cerebellar ataxia; SCA = spinocerebellar ataxia; FRDA = Friedreich’s ataxia; other_ataxias = other ataxias of miscellaneous origin; OR = odds ratio; 95% CI = 95% confidence interval.

## Results

**Fig 1:**
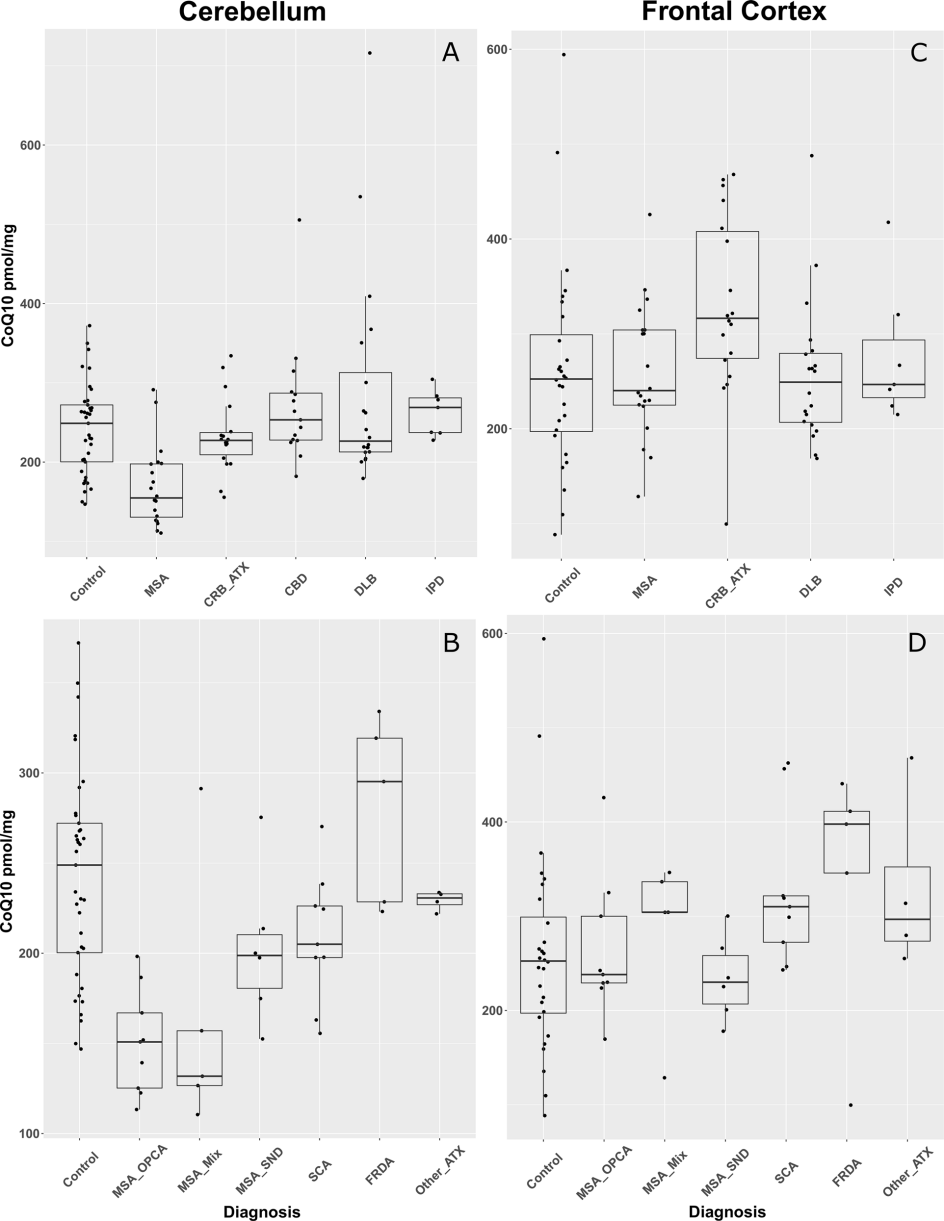
A: Boxplot presenting CoQ10 levels in the cerebellum of MSA, CBD, DLB, IPD, CRB_ATX and controls. B: Boxplot presenting CoQ10 levels in the cerebellum of MSA cases subdivided by pathological subtypes MSA_SND, MSA_mixed and MSA_OPCA, and also CRB_ATX cases subdivided into SCA, FRDA and other_ATX. C: Boxplot presenting CoQ10 levels in the frontal cortex of MSA, DLB, IPD, CRB_ATX and controls. D: Boxplot presenting CoQ10 levels in the frontal cortex of MSA cases subdivided by pathological subtypes MSA_SND, MSA_mixed and MSA_OPCA, and also CRB_ATX cases subdivided into SCA, FRDA and other_ATX. Each dot represents one individual, and dots beyond the boxplot whiskers represent outliers. Glossary: MSA = multiple system atrophy; MSA_SND = MSA striatonigral degeneration; MSA_mixed = MSA mixed; MSA_OPCA = MSA olivopontocerebellar atrophy; CBD = corticobasal degeneration; DLB = dementia with Lewy bodies; IPD = idiopathic Parkinson’s disease; CRB_ATX = cerebellar ataxia; SCA = spinocerebellar ataxia; FRDA = Friedreich’s ataxia; other_ATX = other ataxias of miscellaneous origin.

We measured the CoQ10 levels in brain tissue from the cerebellum and the frontal cortex of MSA, CBD, DLB, IPD, CRB_ATX, and elderly normal controls. Table 1 and Fig 1 show a summary of the results. The analysis of variance revealed an omnibus significant difference between groups for the CoQ10 levels in the cerebellum [F(5,111) = 9.434, P < 0.001], but not in the frontal cortex [F(4,88) = 1.976, NS]. Post hoc pairwise comparisons revealed that this difference is due to lower mean cerebellar CoQ10 levels in MSA when compared to all other diagnosis groups (Tukey test, P ≤ 0.002). The levels of CoQ10 were also mildly reduced in CRB_ATX cases compared to controls (Table 1 and Fig 1), but this did not reach statistical significance.

Following adjustment for potential confounders, we found a significant association between the diagnosis and cerebellar CoQ10 levels (Table 2), with MSA presenting 3% less CoQ10 than controls (Table 2A; OR = 0.97, P = 0.001) and less 3–4% than other disease groups (DLB, IPD, CBD, and CRB_ATX; Table 2C; P ≤ 0.001). Given the findings in the cerebellum, we also subdivided MSA samples into the 3 pathological subtypes, and the CRB_ATX cases into spinocerebellar ataxia (SCA), Friedreich’s ataxia (FRDA) and miscellaneous, and compared these subgroups to controls (Tables 1 and 2B). Significantly lower levels of CoQ10 in the cerebellar tissue were detected only in OPCA (OR = 0.95, P = 0.001) and the mixed type (OR = 0.96, P = 0.005) MSA cases but not in SND cases when compared to controls (Table 2B and Fig 1B). Within the cerebellar diseases (CRB_ATX), even though SCAs presented with the lowest levels of CoQ10, this reduction was still not statistically significant when compared to the control group (Table 2B). In the frontal cortex samples, we found no association between the diagnosis and CoQ10 levels (Table 2D and Fig 1C).

## Discussion

In this study we assessed the CoQ10 levels in the cerebellar and frontal cortices from MSA patients and compared these results to elderly controls and samples from CBD, IPD, DLB and CRB_ATX. This is the first study to have investigated the levels of CoQ10 in a large number of pathologically confirmed MSA brain samples.

The unavailability of basal ganglia tissue as well as the relatively small sample size of some of the disease groups (e.g. IPD) constitutes a limitation of this study. We found, however, significantly decreased levels of CoQ10 in the cerebellar cortex of MSA patients, particularly of the OPCA and mixed pathological subtypes, when compared to controls and all the other disease groups, but no differences were detected in the frontal cortex. Although the role of the *COQ2* variants as a cause of MSA is yet to be replicated, the specific and significant decrease of CoQ10 in the cerebellar cortex of the MSA cohort, but not of cerebellar ataxias (CRB_ATX), suggests that a perturbation in the CoQ10 biosynthetic pathway might be involved in the pathogenesis of MSA. In both cerebellar ataxias and MSA-OPCA and mixed Purkinje cell loss will be present. Our results suggest that although CoQ10 reduction may reflect Purkinje cell loss it is likely that other factors are contributing to the observed effect in MSA. More detailed studies would be required to correlate the degree of Purkinje cell depletion with CoQ10 levels and elucidate the cause and specificity of the CoQ10 biosynthesis impairment in MSA.

CoQ10 related pathways have been previously related to neurodegenerative diseases. The mitochondrial respiratory chain complex I, of which CoQ10 is a cofactor [18, 19], has been found to be dysfunctional in several neurodegenerative diseases, including IPD and progressive supranuclear palsy (PSP). There is increasing evidence that impairment of mitochondrial function and oxidative damage are contributing factors to the pathophysiology of those diseases [20–22]. Furthermore, reduced levels of CoQ10 in cerebral cortex and in lymphocytes of IPD brains have been previously reported [20], and CoQ10 has been also proposed as a biomarker of the antioxidant status in PD [21]. A recent study [23], which measured brain energy metabolism in the basal ganglia of clinically diagnosed MSA-P cases, is not in support of mitochondrial dysfunction playing a primary role in the pathophysiology of MSA. Unfortunately, that study did not include comparisons with the MSA-C clinical subtype to help understanding whether this could be relevant to MSA when the cerebellum is the main affected brain region.

The treatment of MSA is limited and purely symptomatic. Whether the reduction in CoQ10 is linked specifically to the etiology of MSA or is related to the degree of neurodegeneration in the cerebellum of MSA patients is uncertain. However, our results suggest that it may be worth undertaking further studies to evaluate the efficacy of CoQ10 and/or idebenone in the treatment of MSA given that these quinones are reported to be safe and well tolerated in patients [24, 25].

## Conclusion

The role of *COQ2* variants in the etiology of MSA remains debatable. However, our data suggests that a deficiency in cerebellar CoQ10 status may be involved in the pathophysiology of MSA. More work is required before we can elucidate whether this consists of a primary involvement in the pathogenesis of MSA or is a secondary finding due to neurodegeneration.
